# Case Report: Diffuse large B-cell lymphoma presenting predominantly with spinal cord involvement: a series of three cases confirmed by abdominopelvic lymph node biopsy

**DOI:** 10.3389/fnins.2026.1849038

**Published:** 2026-08-26

**Authors:** Yuan Zhu, Hui Liu, Zhong-Hui Zhou, An-Jie Liu, Min Xia

**Affiliations:** 1 Clinical Medical College, Jining Medical University, Jining, China; 2 Department of Neurology, Affiliated Hospital of Jining Medical University, Jining, China

**Keywords:** diffuse large B-cell lymphoma, lymph node biopsy, misdiagnosis, secondary central nervous system lymphoma (SCNSL), secondary spinal cord involvement in lymphoma

## Abstract

**Purpose:**

Secondary central nervous system lymphoma (SCNSL) presenting with predominant spinal cord involvement is rare and often misdiagnosed. This case series aims to describe the clinical characteristics, diagnostic challenges, and the critical role of lymph node biopsy in confirming SCNSL in patients with spinal cord manifestations, with the goal of improving diagnostic accuracy for diffuse large B cell lymphoma (DLBCL) involving the spinal cord.

**Methods:**

We report three cases of SCNSL with predominant spinal cord involvement, all confirmed as DLBCL by abdominopelvic lymph node biopsy. Initial presentations included lower extremity numbness, weakness, and urinary disturbances. Clinical and imaging data were reviewed, along with diagnostic workup and histopathological findings.

**Results:**

All three patients were initially misdiagnosed with myelitis or demyelinating disorders and showed only transient improvement following corticosteroid and immunomodulatory therapy. Spinal MRI revealed abnormal cord signals, while contrast-enhanced abdominal CT and whole-body PET/CT identified hypermetabolic lesions in abdominopelvic lymph nodes. Definitive diagnosis was established by pathological examination of lymph node biopsy specimens, confirming DLBCL.

**Conclusion:**

SCNSL with isolated or predominant spinal cord involvement presents with nonspecific symptoms and can mimic inflammatory myelopathies, leading to diagnostic delays. Lymph node biopsy plays a pivotal role in establishing the diagnosis. Increased awareness of this atypical presentation may facilitate earlier recognition and timely management of DLBCL with spinal cord involvement.

## 1 Introduction

Secondary central nervous system lymphoma (SCNSL) refers to systemic lymphoma involving the central nervous system, among these, cases with spinal cord involvement as the predominant manifestation are particularly rare and are prone to being misdiagnosed as myelitis or demyelinating disorders. The lack of specific clinical manifestations makes early diagnosis challenging, often leading to delayed treatment. In this article, we analyze three cases of diffuse large B-cell lymphoma (DLBCL) involving the spinal cord, all confirmed by abdominal and pelvic lymph node biopsy, and review the relevant literature to investigate the clinical features, diagnostic approach, and role of biopsy in this condition, aiming to inform strategies for early diagnosis in these complex cases.

## 2 Clinical materials

### 2.1 Case 1

A 56-year-old male patient was admitted on January 25, 2024, due to “fever for 30 days and numbness in both lower limbs for 20 days.” The fever began 30 days before presentation, and the patient was diagnosed with an “infectious disease” at a local institution. Although anti-infective treatment was administered, the fever persisted. Twenty days prior to admission, numbness in both feet occurred without an identifiable precipitating factor, and the numbness progressively ascended to the proximal lower extremities. One week before admission, bilateral lower-limb weakness, inability to ambulate, and urinary retention developed. No loss of consciousness, hoarseness, coughing on swallowing, or dysphagia was noted throughout the illness (Detailed pre-hospital data were unavailable). A diagnosis of “myelitis” was made at the local hospital, and pulse methylprednisolone (500 mg for 5 days, 250 mg for 3 days, 120 mg for 3 days, and 60 mg for 4 days) was administered, but without clinical improvement. Subsequently, the patient was transferred to our hospital for ongoing care.

On admission, physical examination revealed that the patient was conscious but in poor spirits, with fluent speech. The water swallowing test yielded normal results. Both pupils were round and equal in size, and the pupillary light reflex was present. Nasolabial folds were symmetric bilaterally, and tongue protrusion was midline. The neck was supple; muscle strength was grade 5 in both upper limbs and grade 3 in both lower limbs; bilateral Babinski signs were negative. Coordination was impaired on heel-knee-shin testing in both lower limbs, and tendon reflexes were reduced in all four limbs. Pinprick sensation was diminished or absent bilaterally below the T12 dermatome.

Following admission, the patient remained febrile and showed little improvement with anti-infective treatment. Laboratory findings on January 25, 2024 showed the following: white blood cell count 3.05 × 10^9^/L, red blood cell count 2.52 × 10^1^^2^/L, hemoglobin 80 g/L, and platelet count 172 × 10^9^/L. Cerebrospinal fluid (CSF) analysis on February 2, 2024 revealed: CSF white blood cells 6 × 106/L, CSF protein 0.93 g/L, glucose 1.81 mmol/L, chloride 1.09 mmol/L, adenosine deaminase 0.7 U/L, CSF IgA 45.90 mg/L, CSF IgG 199.00 mg/L, CSF IgM 3.00 mg/L, and CSF microalbumin 624.00 mg/L. Both serum and CSF tested negative for AQP4, MOG, MBP, and oligoclonal bands. Bone marrow aspiration and cytology performed on January 28, 2024 from the sternum showed increased and coarse cytoplasmic granules in the granulocytic series, evidence of hemophagocytosis, and megaloblastic changes in some cells across all three lineages; iron staining of the bone marrow revealed deficient intracellular iron.

**Figure 1:**
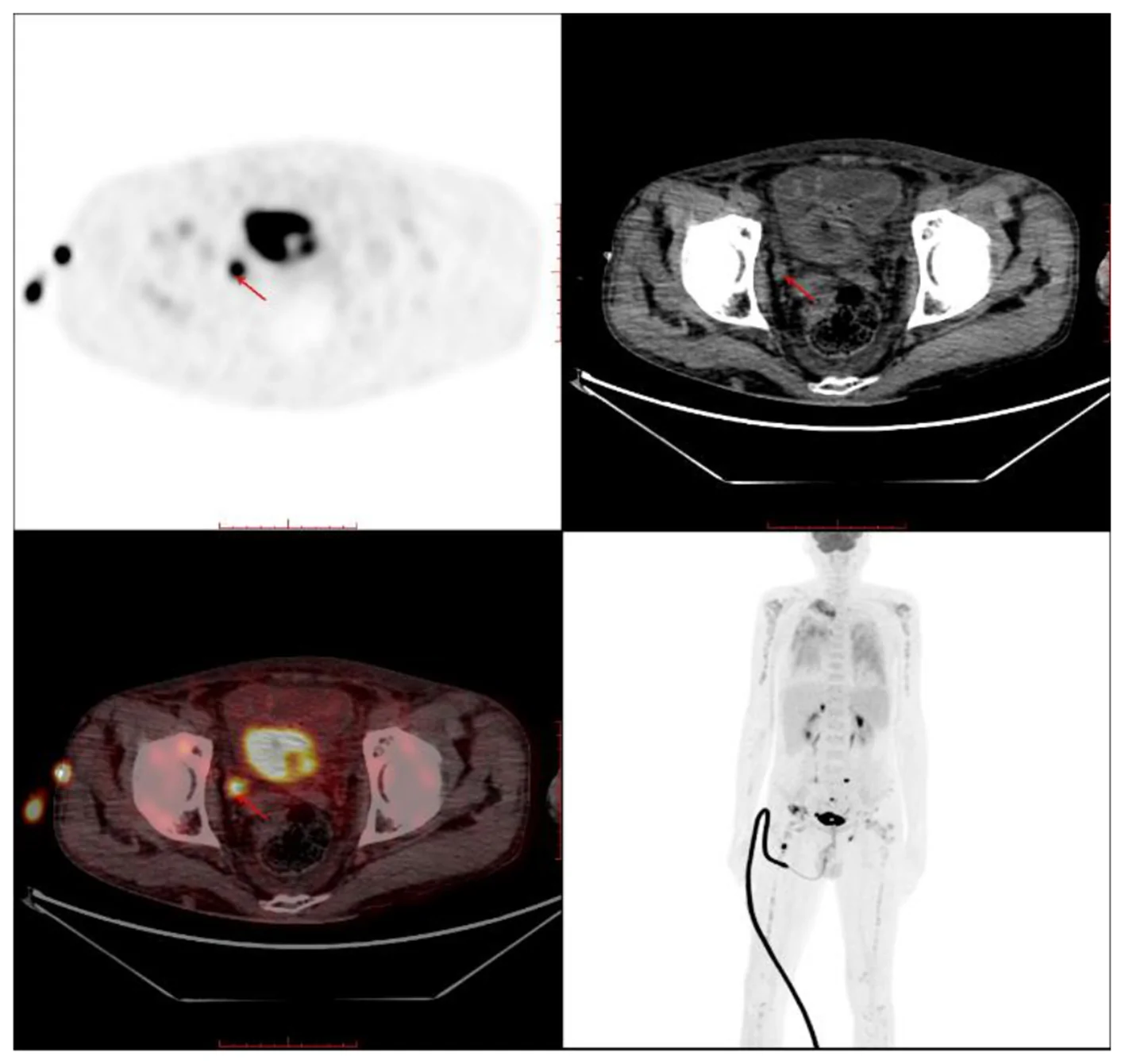
PET imaging revealed enlarged lymph nodes in the right pelvic region with increased metabolic activity.

Supplementary investigations were performed on January 26, 2024: unenhanced and contrast-enhanced brain MRI showed chronic lacunar infarcts involving the bilateral basal ganglia and corona radiata. MRI of the thoracic and lumbar spine demonstrated mild posterior disc protrusion at T4-5 and suspected yellow marrow conversion involving the T1 through T12 vertebral bodies. A PET/CT scan of the whole body on January 29, 2024 (Figure 1) showed: multiple foci of increased metabolism were observed in the bones throughout the skeleton, with corresponding regions of increased bone density in some areas; nodules in the retroperitoneal region adjacent to the right adrenal gland and enlarged lymph nodes in the right pelvis also exhibited hypermetabolism. Based on these findings, a diagnosis of lymphoma was suspected. Consequently, further evaluation with ultrasound-guided core needle biopsy of the right pelvic lymph node was carried out on February 3, 2024 to establish a definitive pathological diagnosis.

Pathological examination of the lymph node biopsy on February 6, 2024 revealed the following: immunohistochemical staining demonstrated tumor cells that were diffusely positive for CD20 and CD79a, positive for CD5, negative for CD21, CD10, CD56, EMA, ALK, and CD30, scattered positivity for Bcl-6, positive for MUM-1, positive for C-myc (approximately 30%), positive for P53 (40%−50%), with T cells positive for CD2, CD3, and CD7, and a Ki-67 proliferation index >75%. These findings were consistent with a non-Hodgkin B-cell lymphoma, specifically diffuse large B-cell lymphoma of the non-germinal center B-cell subtype.

Following confirmation of the diagnosis, the patient was discharged at the family's request. The patient's condition progressed rapidly with acute deterioration, leading to death on May 1, 2024.

### 2.2 Case 2

A 58-year-old male patient. He was first admitted on February 3, 2022, due to “right lower limb weakness accompanied by urinary retention for 10 days.” More than 10 days prior to admission, the patient developed right lower limb weakness without obvious triggers, resulting in inability to walk with the right leg; this was accompanied by urinary retention and a sensation of numbness below the abdomen, which persisted without relief.

On admission, physical examination revealed that the patient was conscious, in stable mental condition, and able to speak fluently. Both pupils were equal and round, approximately 3 mm in diameter, with prompt light reflexes, and extraocular movements were intact. The neck was supple; nasolabial folds were symmetric bilaterally; tongue protrusion was midline. Muscle strength was 3/5 in the right lower extremity and 5/5 in the remaining limbs, with normal tone throughout. Bilateral Babinski signs were negative, and no sensory deficits were detected. The heel-knee-shin test was unstable on the right.

**Figure 2:**
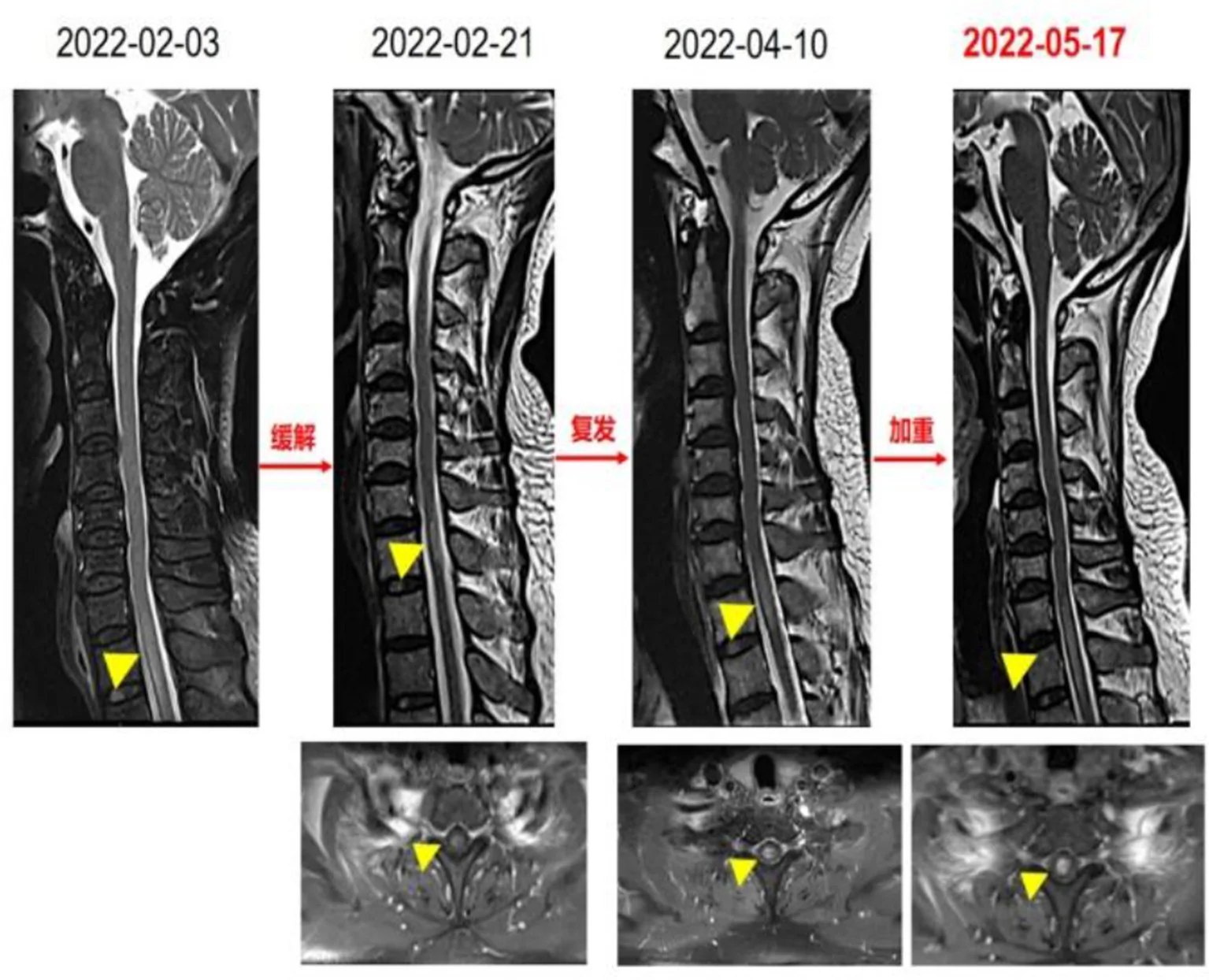
The figure presents cervical and thoracic MR images demonstrating abnormal spinal cord signal at the C7-T1 level, along with the changes in this signal over multiple treatment episodes.

Serial cerebrospinal fluid (CSF) analyses were performed throughout the patient's clinical course (Table 1). Serum and CSF testing on February 15, 2022, revealed negative results for AQP4, MOG, MBP, and oligoclonal bands. Plain and contrast-enhanced cervical and thoracic spine MRI on February 4, 2022 (Figure 2) showed abnormal intramedullary signal at the C7-T1 vertebral level, suggestive of inflammation. The patient was treated with pulse methylprednisolone (0.5 g). Repeat unenhanced and contrast-enhanced MRI of the cervical and thoracic spine on February 21, 2022 (Figure 2) revealed a reduction in the extent of abnormal intramedullary signal at the C7-T1 level relative to the prior examination; the patient was discharged on February 22, 2022.

**Table 1:** Cerebrospinal fluid findings during the clinical course of case 2.

CSF	2022-02-04	2022-04-09	2022-05-17	Normal reference range
cerebrospinal fluid pressure (mmH_2_O)	102	140	180	80–180
physical characteristics	Colorless an d clear	Colorless and clear	Colorless and clear	
WBC ( × 10^6^/L)	2	3	1	0–5
glucose (mmol/L)	3.21	2.37 **↓**	3.98	2.5–4.5
Pandy's test	Negative	Weakly positive	Positive (1+)	
protein (g/l)	0.74**↑**	1.21**↑**	1.9**↑**	0–0.4
IgA (mg/l)	4.37	7.76**↑**	13.1 **↑**	0–5
IgG (mg/l)	76.6**↑**	100**↑**	219**↑**	0–30
IgM (mg/l)	0.55	1.92↑	5.67↑	0–1.3
microalbumin (mg/l)	472↑	969↑	1290↑	0–350

The meaning of “↑” is that the result levels above the maximum of the standard range. The meaning of “↓” is that the result levels Below the minimum of the standard range.

The patient was readmitted on April 8, 2022, for the second hospitalization, presenting with “recurrent worsening of right limb weakness over 2 weeks and a constricting band sensation around the waist.” A PET-CT conducted on April 8, 2022, showed no hypermetabolic neoplastic lesions anywhere in the body. A repeat lumbar puncture was performed on April 9, 2022 (Table 1). Flow cytometric examination revealed that 784 nucleated cells were collected. (1) No tumor cells were identified. (2) The cell population was predominantly composed of T lymphocytes, with no obvious aberrant immunophenotype. Cervical and thoracic spine MRI with contrast on April 10, 2022 (Figure 2) revealed abnormal intramedullary signal with enhancement at the C7-T1 level, with a relatively increased extent compared with previous imaging. Immunomodulatory therapy consisting of intravenous immunoglobulin, prednisone acetate, and mycophenolate mofetil dispersible tablets was administered, and the patient was transferred to the rehabilitation department on April 23, 2022. Following discharge, regular treatment with prednisone acetate and mycophenolate mofetil dispersible tablets was continued.

The patient was hospitalized for the third time on May 17, 2022, presenting with “one week of progressive numbness and weakness in both lower extremities, along with blurred vision in the right eye and abdominal distension.” A repeat CSF examination was performed on May 17, 2022 (Table 1); contrast-enhanced MRI of the cervical and thoracic spine (Figure 2) demonstrated abnormal intramedullary signal at the C7-T2 vertebral level, suggestive of myelitis (showing increased lesion extent but decreased enhancement intensity compared with previous images). Therapy with plasma exchange and ofatumumab was administered. The patient experienced mild symptom relief and became capable of ambulating short distances. Contrast-enhanced MRI of the cervical and thoracic spine on June 24, 2022 (Figure 2) revealed no interval change in the lesion.

**Figure 3:**
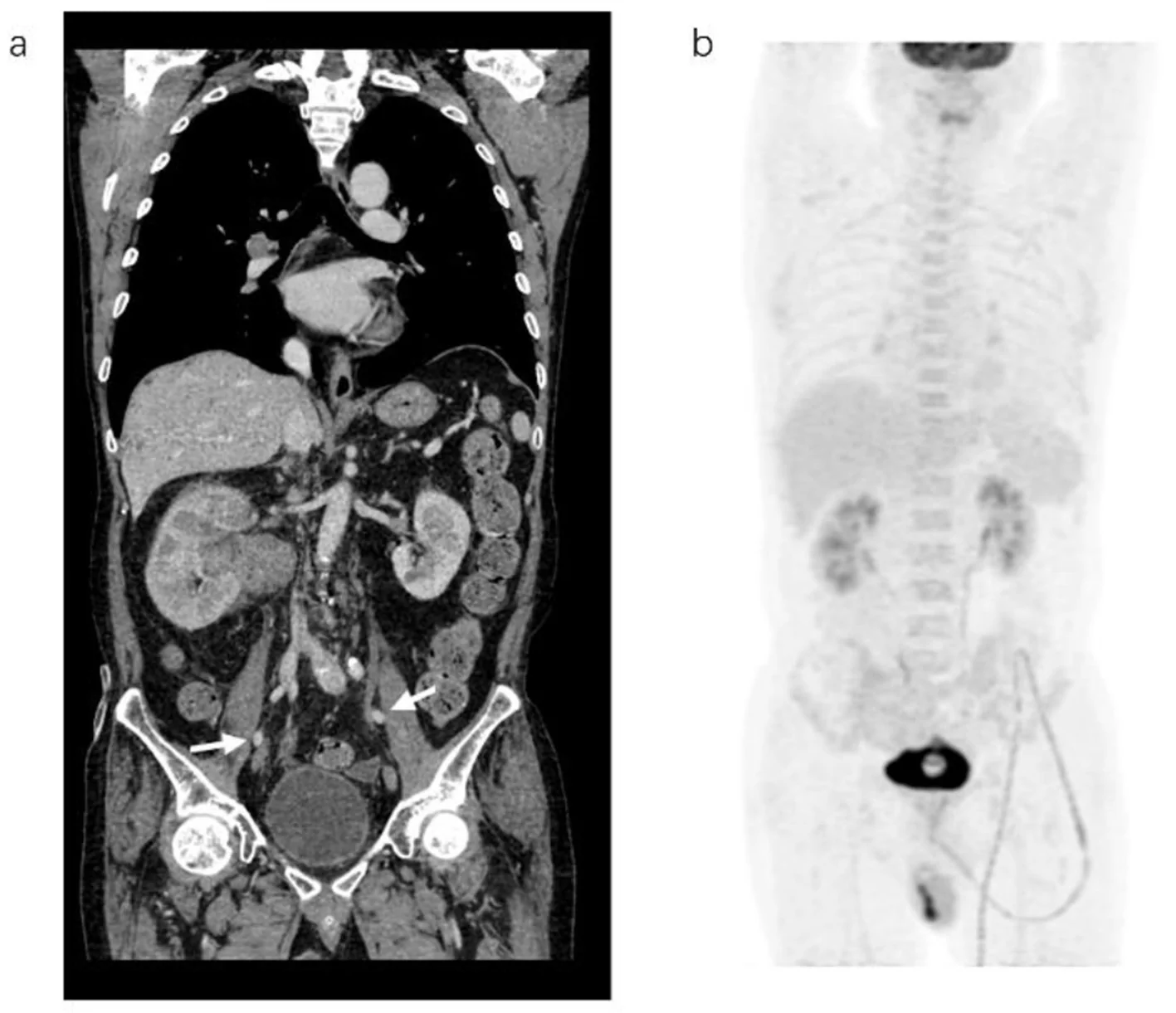
**(a)** An abdominal CT image of the patient, demonstrating multiple enlarged lymph nodes in the retroperitoneal and pelvic regions. **(b)** A PET image of the patient, showing no evidence of hypermetabolic neoplastic lesions.

The patient was hospitalized for the fourth time on November 10, 2022, presenting with “one week of aggravated numbness and weakness in both lower extremities, along with marked abdominal distension and a constricting band sensation involving the chest and back.” A repeat lumbar puncture was performed on November 20, 2022 (Table 1). Plain and contrast-enhanced CT of the whole abdomen and chest on November 23, 2022 (Figure 3) revealed a soft tissue nodule adjacent to the right ureter near the bladder, marked thickening of the left posterior bladder wall, and enlarged lymph nodes in the right hilum, retroperitoneum, and pelvis; findings were suggestive of a malignant neoplasm. Transrectal prostate ultrasound on November 25, 2022 showed a hypoechoic mass in the left pelvic region adjacent to the bladder wall, suggestive of a space-occupying lesion. A biopsy of the perivesical mass was performed on November 26, 2022. Histopathology of the mass demonstrated the following: immunohistochemistry showed: CD20 (+), CD79a (+), CD10 (–), Mum-1 (–), Bcl-2 (+), Bcl-6 (+), CD30 (partial +), C-myc (–), CD21 (–), CD3 (T cells +), Cyclin D1 (occasional +), Vimentin (+), GATA-3 (–), PAX-5 (+), CK (–), PSA (–), Ki-67 (+, 70%−80%); *in situ* hybridization showed EBER (–). The findings were consistent with a lymphoproliferative disorder, and in combination with the immunohistochemical results, the diagnosis was supported as aggressive B-cell lymphoma, specifically diffuse large B-cell lymphoma (germinal center B-cell type). The patient was transferred to the hematology department on November 27, 2022, where bone marrow smear and flow cytometry revealed no obvious abnormalities.

Following a confirmed diagnosis of non-Hodgkin lymphoma (diffuse large B-cell lymphoma) and after excluding contraindications to chemotherapy, the patient received treatment with the R-CDOP regimen plus zanubrutinib. Thereafter, the patient experienced recurrent episodes of fever, abdominal distension, and jaundice, and anti-infective treatment was given. In February 2023, the patient developed abdominal pain and distension. On February 22, 2023, MRCP together with contrast-enhanced MRI of the upper abdomen demonstrated heterogeneous thickening involving the intra- and extrahepatic bile ducts, the common bile duct, and the gallbladder wall, along with multiple nodular lesions in the right perinephric space, renal pelvis, and right paraspinal area, raising suspicion of lymphomatous involvement. Laboratory findings on February 21, 2023 revealed total bilirubin of 150.1 μmol/L and direct bilirubin of 134.2 μmol/L. Percutaneous transhepatic biliary drainage was recommended, but the patient's family declined the procedure. Chemotherapy consisting of the CP regimen plus mitoxantrone, selinexor, and orelabrutinib was then administered. The patient continued to have intermittent abdominal pain and distension. No response to treatment was observed, and the patient died in April 2023.

### 2.3 Case 3

A 65-year-old man was initially hospitalized on May 25, 2023, presenting with “numbness and weakness in the left lower extremity for 3 months, aggravated during the preceding two weeks.” Three months prior to admission, the patient developed low back pain and discomfort without obvious triggers, accompanied by numbness and weakness in the left lower limb, occasional falls, and difficulty with defecation and occasional straining during urination. Two weeks before admission, the left lower-limb numbness and weakness progressed to the point where he was unable to ambulate independently. Ten days before admission, numbness appeared in the right lower extremity. Throughout the disease course, movement of both upper limbs remained normal. He had not received systematic evaluation or treatment prior to admission, and the symptoms persisted without improvement, prompting him to seek care at our hospital.

On admission, physical examination revealed an alert patient in fair mental status with fluent speech. The pupils were equal and round, measuring approximately 3 mm, with brisk light reflexes. Ocular motility was full in all directions, and no nystagmus was observed. The nasolabial folds were symmetrical bilaterally, and the tongue protruded in the midline. Muscle strength was 2/5 in the left lower extremity and 4/5 in the right, with normal tone throughout. The babinski sign was negative on the left and positive on the right. Bilateral knee reflexes were reduced, and pain sensation was intact without obvious deficits.

On May 25, 2023, a lumbar puncture yielded an opening pressure of 180 mmH_2_O. CSF analysis demonstrated a Pandy test result of positive (+). CSF biochemical analysis showed lactate dehydrogenase at 46 U/L and total CSF protein at 0.918 g/L. The CSF specific protein panel demonstrated CSF IgA 28.80 mg/L, CSF IgG 124.00 mg/L, CSF IgM 8.68 mg/L, and CSF microalbumin 595.00 mg/L; findings suggestive of an immune-mediated process. Both serum and CSF tested negative for AQP4, MOG(IgG), MBP, and oligoclonal bands. Laboratory tests for ASO, RF, and CRP on May 25, 2023, revealed β2-microglobulin of 3.56 mg/L and C-reactive protein of 17.78 mg/L, indicating an activated immune response; other results were within normal limits. The anemia workup showed a folic acid level of 2.6 ng/mL.

**Figure 4:**
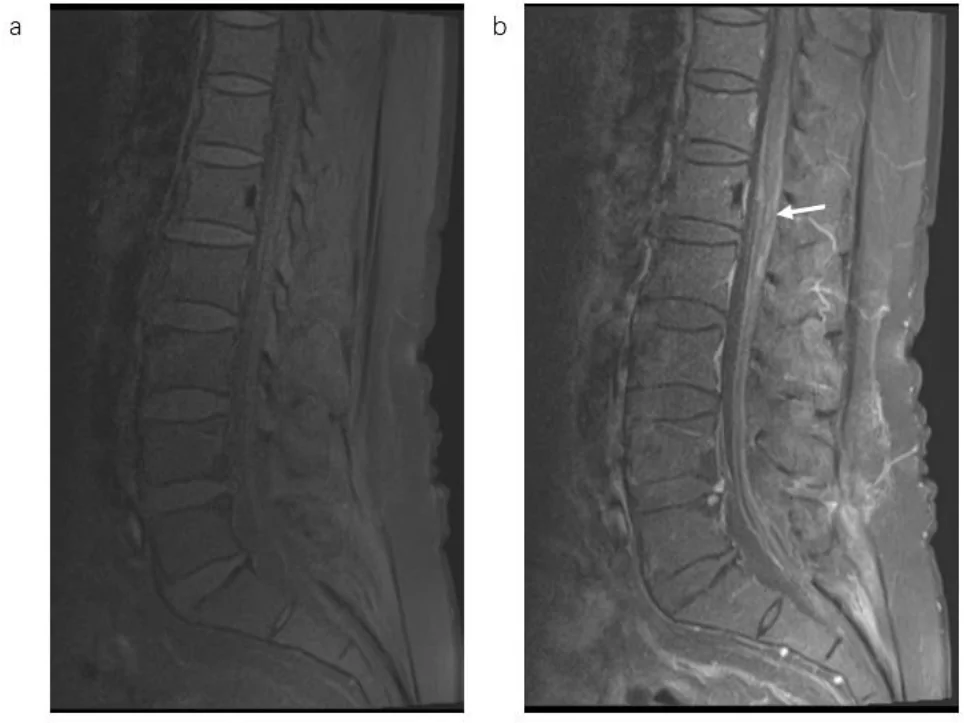
**(a, b)** T1-weighted and contrast-enhanced T1-weighted MR images of the patient's spinal cord, respectively, demonstrating mild linear and patchy enhancement involving the conus medullaris.

Lumbar and thoracic spine MRI on May 27, 2023 (Figure 4) showed the following: (1) Mild linear and patchy enhancement of the conus medullaris—inflammatory lesion to be excluded; (2) Mild enhancement of soft tissue posterior to the left lamina at L4-5, possible inflammatory lesion; (3) Thoracolumbar degeneration and subcutaneous fasciitis in the lumbodorsal region; (4) Abnormal signal in the left kidney. On May 27, 2023, given the persistence of pronounced limb numbness, the oral pregabalin dose was escalated, and pulse methylprednisolone (0.5 g) was initiated. Following treatment, the patient's symptoms improved, and he was discharged on June 10, 2023.

**Figure 5:**
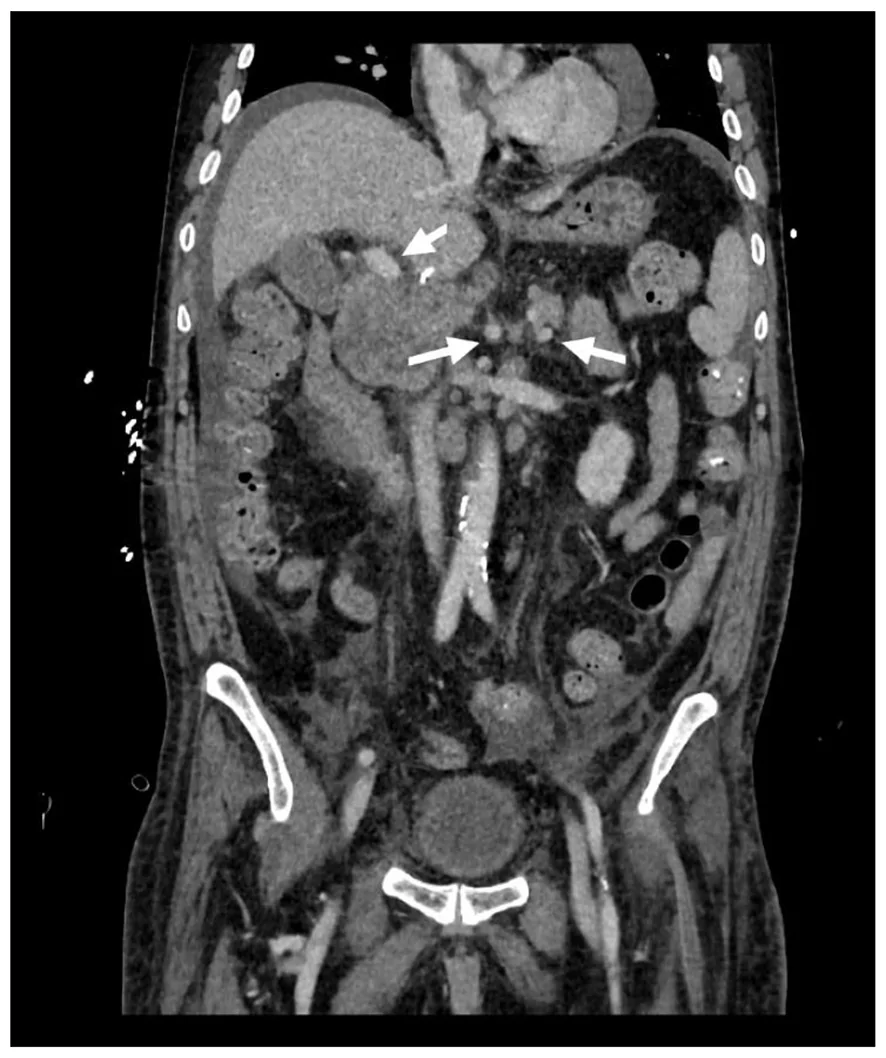
The figure shows the patient's abdominal CT, revealing multiple enlarged lymph nodes in the hepatic portal region and retroperitoneum.

**Figure 6:**
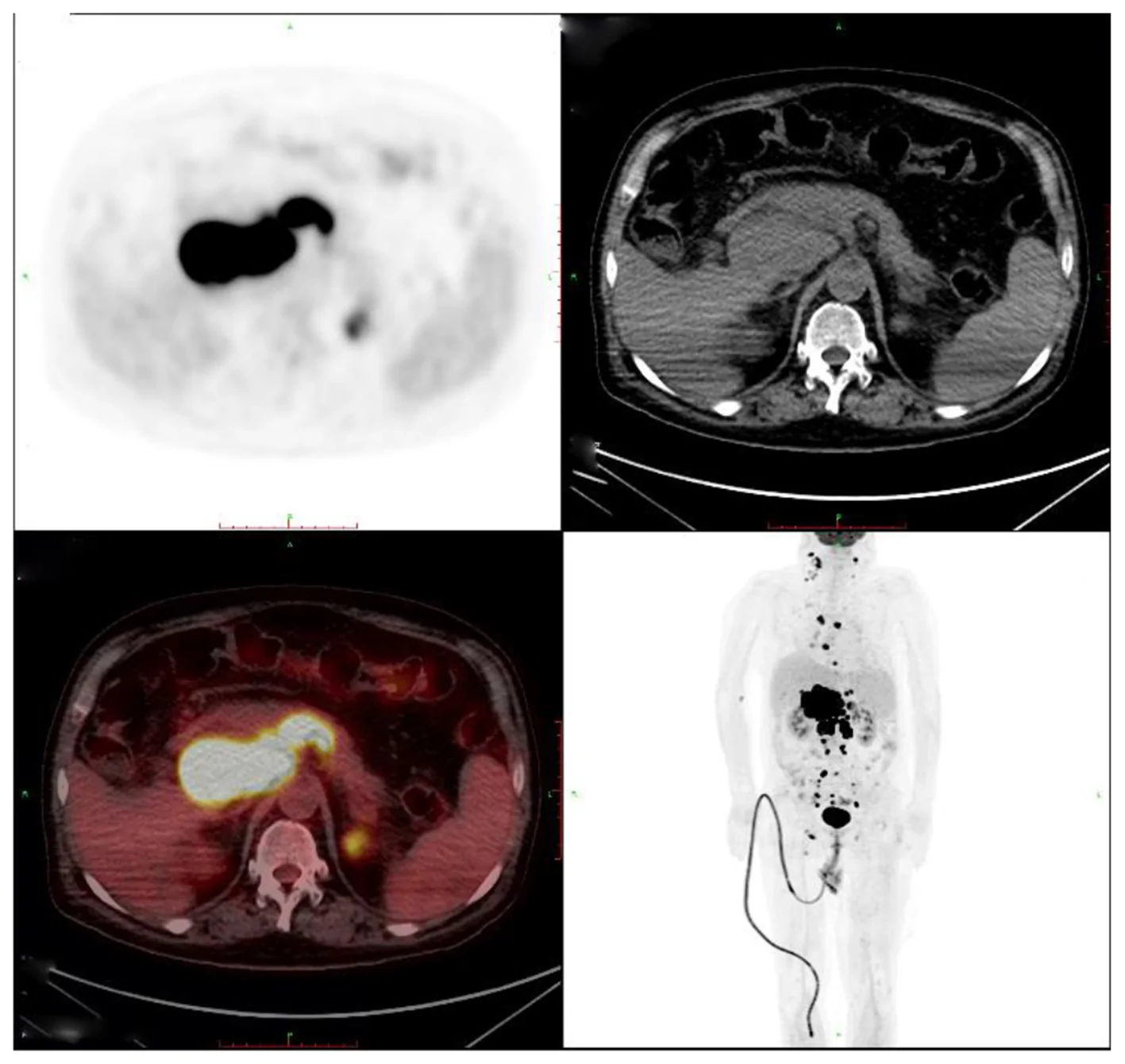
PET scan demonstrated multiple hypermetabolic lymph nodes, raising strong suspicion of lymphoma.

On July 11, 2023, the patient was hospitalized for a second admission, presenting with “recurrent fever lasting over one month.” Abdominal ultrasound on July 16, 2023 revealed multiple hypoechoic nodules and masses in the hepatic portal region and retroperitoneum. The patient developed abdominal distension and pain. On July 17, 2023, abdominal CT (Figure 5) revealed multiple low-density mass lesions in the hepatic portal region and retroperitoneum, consistent with lymphadenopathy, as well as ascites and pelvic fluid. On July 23, 2023, ultrasound-guided catheter drainage of ascites was carried out. A PET/CT of the whole body on July 24, 2023 (Figure 6) demonstrated: (1) multiple hypermetabolic lymph nodes in the areas described above, strongly suggestive of lymphoma, along with a hypermetabolic nodule at the left kidney upper pole, also concerning for lymphomatous infiltration; (2) mildly increased metabolism in bilateral hilar and remaining mediastinal lymph nodes, likely due to inflammatory hyperplasia; and (3) a hypermetabolic nodule at the edge of the left lobe of the liver.

Pathological examination of the retroperitoneal mass biopsy on July 27, 2023 revealed: (lymph node biopsy tissue) aggressive B-cell lymphoma; based on HE morphology and immunohistochemistry, the findings favored diffuse large B-cell lymphoma (non-germinal center subtype). Molecular testing was recommended to rule out high-grade B-cell lymphoma. Immunohistochemistry showed: CD20 (+), CD79a (+), CD3 (T cells +), CD21 (–), Bcl-6 (–), Bcl-2 (+), CD10 (–), Mum-1 (+), Cyclin D1 (–), c-myc (3+, 30%), Ki-67 (+, 80%), CK (–); *in situ* hybridization showed: EBER (–). The diagnosis of aggressive B-cell lymphoma was confirmed. The patient received chemotherapy using the CDOP regimen, with adjunctive antiemetic therapy and gastric mucosal protection.

The patient underwent a total of seven hospitalizations in the hematology department at our institution. A recent whole-body PET/CT scan performed on October 21, 2025, following chemotherapy for DLBCL, was compared with the PET/CT study from April 17, 2024. The findings demonstrated: slightly enlarged lymph nodes in the mediastinum adjacent to the right trachea and along the left iliac vessels, which remained stable in size with mild to moderately increased FDG uptake; close surveillance was advised for multiple small lymph nodes in the bilateral cervical region, the remainder of the mediastinum, the abdominopelvic cavity, and the retroperitoneum, none of which exhibited abnormal hypermetabolism. The nodule at the upper pole of the left kidney was unchanged in size with no significant abnormal metabolic activity, suggesting loss of biological activity or metabolic suppression of the lesion. Multiple enlarged or small lymph nodes in the bilateral inguinal region with identifiable hilar structures were stable in size and showed mild hypermetabolism, likely representing inflammatory lymph nodes. After a follow-up period of 32 months, the patient died.

## 3 Discussions

Lymphoma originates in the lymph nodes and lymphoid tissues. As a malignancy of the immune system, it may disseminate to the central nervous system (CNS), primarily involving the brain, spinal cord, and peripheral nerves. Lymphoma manifesting predominantly with spinal cord involvement is extremely uncommon (Fan et al., 2023), representing fewer than 1% of cases of primary central nervous system lymphoma (PCNSL). Owing to the nonspecific nature of the clinical symptoms, histopathological biopsy is considered the diagnostic gold standard. However, due to the invasiveness of biopsy procedures and the interference of glucocorticoid treatment, the number of cases definitively diagnosed via spinal cord biopsy in real-world settings remains considerably low, making the diagnosis of SCNSL very difficult. In this context, the relevant literature (Gao, 2021) suggests that patients presenting with intramedullary spinal cord lesions on MRI and a histopathologically confirmed history of or synchronous lymphoma should be regarded as having intramedullary spinal cord metastasis of lymphoma.

Thus far, only a limited number of published studies have addressed SCNSL involving the spinal cord. Flanagan et al. (2012) reported 7 cases of non-Hodgkin lymphoma (NHL) with spinal cord involvement admitted to the Mayo Clinic between 1996 and 2010, of which 5 cases represented secondary spinal cord involvement from systemic lymphoma. The median time from diagnosis of systemic NHL to spinal cord involvement was 8 months. Two cases presented initially with myelopathy and were diagnosed by bone marrow biopsy. Among the cases, 3 were diffuse large B-cell lymphoma (DLBCL), 2 were follicular B-cell NHL (grades 2 and 3, respectively), 1 was lymphoplasmacytic NHL, and 1 was peripheral T-cell NHL (not otherwise specified). Cerebrospinal fluid (CSF) analysis performed in 6 patients revealed pleocytosis (4/6), elevated protein (6/6), hypoglycorrhachia (1/6), and positive CSF cytology for lymphoma cells (4/6). Spinal MRI in all 7 patients demonstrated T2 hyperintensity (7/7), with involvement of the cervical cord (3/7) and thoracic cord (3/7). PET imaging revealed hypermetabolic lesions in the spinal cord in 2 patients. Six patients were followed up, and all had died. The median survival from the diagnosis of intramedullary NHL to death was 11.5 months. The Kaplan-Meier survival estimate indicated a 50% survival rate at 15 months.

An analysis of demographic characteristics in this study showed a higher incidence of the disease in males compared to females, with all three patients being male. This is consistent with a literature review that identified male predominance (6/8 cases) (Hara et al., 2024). The mean age at onset in this study was 60 years (range 56–65), which is consistent with the previously reported mean age of 61 years (range 41–81). In terms of clinical symptoms, patients presented with lower extremity numbness and weakness, urinary retention, fever (which could appear either at an early or late stage), and abdominal distension or pain. None of the three patients in this study had upper limb symptoms, in Case 2, the lesion was located in the spinal cord at the C7-T1 vertebral level. The patient had normal upper limb strength but right lower limb weakness, a pattern suggesting involvement of the lateral corticospinal tract; in contrast, the anterior horn cells at this level were spared. Given that the presentation of spinal cord involvement is frequently nonspecific and prone to misdiagnosis as acute myelitis among other disorders, imaging examination is therefore essential in the differential diagnostic workup.

Spinal lymphoma lesions predominantly affect the lower cervical spinal cord, upper thoracic spinal cord, lumbosacral nerve roots, and cauda equina, with perilesional edema observed in over 90% of cases (Marquardt and Li, 2018). MRI findings commonly demonstrate isointensity or hypointensity on T1-weighted images and isointensity or hyperintensity on T2-weighted images, with contrast-enhanced imaging typically revealing pronounced lesion enhancement that can persist beyond 2 months (Nakamizo et al., 2002; Küker et al., 2005). A comparative study evaluating MRI characteristics of spinal cord lymphoma and NMOSD revealed that while both diseases may exhibit T2 hyperintensity and contrast enhancement in the cervical spinal cord, persistent enhancement lasting over 2 months was found in 100% of spinal cord lymphoma cases vs. only 12% of NMOSD cases. Furthermore, cauda equina involvement occurred in 57% of spinal cord lymphoma patients but was not observed in NMOSD. Together, these imaging features represent critical discriminators between spinal cord lymphoma and NMOSD (Flanagan et al., 2011). Most lesions on PET-CT exhibit homogeneous increased fluorodeoxyglucose (FDG) avidity. However, a subset of patients may have unremarkable PET-CT findings, possibly related to a longer disease duration, indolent tumor cell proliferation, and involvement of the spinal dura mater, a structure that typically demonstrates low metabolic activity (Pons-Escoda et al., 2023).

In this study, all three cases had normal CSF cell counts, mildly elevated total protein levels, mildly decreased glucose and chloride levels, and increased CSF IgA and IgG levels. Ikeguchi et al. (2018) reported that the median CSF cell count in the CNS lymphoma group was significantly higher than that in the glioma and multiple sclerosis (MS) groups (*p* < 0.001), and the median CSF protein level was significantly higher than that in the MS and neuromyelitis optica spectrum disorder (NMOSD) groups. However, such changes are nonspecific. Previous studies have further confirmed that CSF levels of IL-10 and sIL-2R are elevated in patients with primary intramedullary spinal cord lymphoma (PISCL), and these are considered effective biomarkers for the diagnosis of PISCL (Ikeguchi et al., 2018). In terms of blood tests, all three patients in this study had elevated serum lactate dehydrogenase (LDH) levels and elevated erythrocyte sedimentation rates (ESR). Lactate dehydrogenase (LDH) is a glycolytic enzyme ubiquitously distributed in human tissues, and its synthesis is upregulated following malignant transformation of normal tissues (Bitencourt et al., 2026). Previous research has indicated that a dynamically increasing LDH level constitutes an independent risk factor for recurrence of diffuse large B-cell lymphoma (DLBCL).

The three cases described herein all presented initially with limb numbness or weakness, with no distinct clinical specificity. The primary features were consistent with spinal cord involvement, and all were initially misdiagnosed as myelitis or demyelinating disorders. Following treatment with corticosteroids and immunomodulatory agents, one patient (1/3) showed a reduction in the lesion extent on follow-up MRI, which added a layer of complexity to the diagnostic process. Throughout the disease course, the patients exhibited relapsing episodes, accompanied by urinary retention, recurrent abdominal distension, and fever. Abdominal CT imaging identified multiple enlarged lymph nodes and nodular masses within the abdominal cavity, while PET-CT showed hypermetabolic activity in these lesions. Ultimately, all cases were definitively diagnosed as diffuse large B-cell lymphoma (DLBCL) by lymph node biopsy.

DLBCL has been shown to affect the spinal cord, presenting either as an intramedullary or extramedullary process. For intramedullary lesions, the clinical manifestations are diverse, often presenting with progressive limb weakness, sensory disturbances, and bladder and bowel dysfunction (Kitahara et al., 2023; Doi et al., 2025); additionally, some patients may also develop involvement of the conus medullaris. MRI usually reveals longitudinally extensive T2 hyperintense lesions that may extend from the medulla to the thoracic cord (Miura et al., 2021). On contrast-enhanced imaging, the lesions may enhance homogeneously, but this feature lacks specificity. In contrast, for extramedullary disease, pain at different locations is the main presenting symptom (Cunha et al., 2022), followed over time by progressive limb weakness, sensory impairment, and autonomic dysfunction (Lapolla et al., 2024). MRI may demonstrate various patterns, including isointense, homogeneous epidural contiguous soft-tissue masses and expansile osteolytic lesions (Shu et al., 2019). Despite the generally dismal prognosis for both types, prompt treatment may nevertheless lead to a relatively favorable outcome.

DLBCL represents the most frequent type of non-Hodgkin lymphoma in adults, comprising 35% to 50% of all non-Hodgkin lymphoma cases in China (Wang, 2023). The typical manifestation of this disease is rapidly progressive lymphadenopathy, and most patients are diagnosed and managed by hematologists. However, a subset of patients present initially with neurological manifestations and are first evaluated by neurologists, with clinical characteristics and laboratory findings mimicking those of multiple neurological conditions, including cerebrovascular disease, demyelinating disorders, and viral encephalitis. In this study, all three patients had a pathological diagnosis of DLBCL, yet their initial diagnoses were all spinal cord demyelinating disorders; case 2 required 296 days from the initial presentation to reach a confirmed diagnosis. In clinical management, the possibility of SCNSL should be suspected in patients who exhibit spinal cord symptoms along with systemic features including fever, abdominal pain, distension, anemia, and recurrence despite immunotherapy. In such cases, abdominal imaging with CT or PET-CT should be conducted to detect enlarged lymph nodes or nodular masses, and timely biopsy should be performed to achieve early definitive diagnosis, facilitating prompt targeted therapy and potentially improving survival outcomes.

The lack of spinal cord tissue biopsy in the present study represents a limitation of this case series; however, it is also consistent with real-world clinical practice. While spinal cord biopsy is the gold standard for diagnosing spinal cord lesions, it is performed only in selected special circumstances (e.g., sarcoidosis) in actual clinical practice. This is due to several factors: the high risk of spinal cord functional impairment, the fact that imaging diagnosis is sufficiently accurate in most cases, and the possibility of diagnosing many cases on clinical grounds alone.

## Data Availability

The original contributions presented in the study are included in the article/supplementary material, further inquiries can be directed to the corresponding author.
